# Exploring Barriers Toward Telehealth in an Underserved, Uninsured Patient Population

**DOI:** 10.1089/tmr.2024.0036

**Published:** 2024-08-19

**Authors:** Ashna Raiker, Meenu Johnkutty, Ambar Ruiz, Jedan Phillips, Melissa J. Earle

**Affiliations:** ^1^ Renaissance School of Medicine, Stony Brook University, Stony Brook, New York, USA.; ^2^ Department of Family, Population, and Preventative Medicine, Stony Brook University Hospital, Stony Brook, New York, USA.; ^3^ Stony Brook School of Social Welfare, Stony Brook University, Stony Brook, New York, USA.

**Keywords:** telehealth, uninsured, COVID-19, health care disparities

## Abstract

**Background:**

Telehealth has untapped potential to improve health care for underserved communities. However, it remains underutilized, limiting opportunities to improve continuity of care and health care outcomes. This pilot study investigates attitudes and barriers to telehealth at Stony Brook HOME, Renaissance School of Medicine’s student-run free-health clinic in Suffolk County, NY.

**Methods:**

Surveys (*n* = 100) were electronically administered bimonthly during clinic waiting room time from May 2022 to August 2023 in both English (40%) and Spanish (60%). Surveys collected information on patient demographics, perceived patient barriers and attitudes to telehealth, and technological comfort levels.

**Results:**

Most patients were Hispanic/Latino (68%), female (54%), and 40–60 years old (52%). Spanish speakers often come from high social vulnerability regions. English speakers were more likely to own a smartphone, computer, or tablet than Spanish speakers (*p* = 0.046). English speakers reported higher levels of technological comfort using a smartphone or tablet (*p* = 0.0033) and using it for their health care (*p* = 0.03). Finally, 100% of English speakers reported reliable internet access compared to 66.7% of Spanish speakers.

**Discussion:**

These results demonstrate that barriers to telehealth are being disproportionately felt by Spanish speakers, thus necessitating survey-directed interventions to address this disparity.

## Introduction

Telehealth has untapped potential to improve health care for underserved communities. It can increase continuity of care for patients with chronic health conditions, reduce transportation and babysitting costs, reduce work absences, and decrease the stress and time drain associated with waiting rooms.^1–4^ However, barriers that limit access to quality virtual care persist in socioeconomically vulnerable communities.^2^ These barriers include but are not limited to a lack of technological literacy, language barriers, and access to devices.^2^ Without identifying and addressing these barriers to access telemedicine, health care inequities will continue to persist in these communities, increasing morbidity and mortality.^5^

Free student-run clinics have been invaluable to socioeconomically disadvantaged communities.^6^ Since its founding in 2007, Stony Brook HOME (SB HOME), a medical student-run clinic in Patchogue, NY, has provided free preventive medical care, nutritional counseling, and social work to the uninsured in Suffolk County. Patients at SB HOME frequently come from neighboring communities such as Brentwood and Central Islip, two areas with high social vulnerability indices, as defined by the Centers for Disease Control and Prevention. Low socioeconomic status, aggregate housing, disability, minority status and language, and housing type and transportation are factors that contribute to the high social vulnerability found in SB HOME’s patient population.^7^

Considering Centers for Disease Control and Prevention (CDC) social distancing protocols, in-person visits to SB HOME were significantly reduced at the start of the pandemic, and a new telemedicine program was launched in May 2020 to facilitate virtual visits. A cohort of SB HOME patients received smartphones under grant funding from the COVID-19 Telehealth Program, an initiative started by the Federal Communications Commission. However, since the telemedicine program’s inception, only 13% of all clinic encounters between January 2020 and February 2022 were telemedicine visits, and many did not seek follow-up appointments. This underutilization warranted further exploration as to why telehealth is not a popular option. Thus, this study aimed to explore the underlying attitudes and barriers to telehealth in our patient demographic. Survey-based interventions were designed to address and overcome these barriers, ultimately with the goal of improving SB HOME’s telehealth infrastructure by addressing health inequity.

## Materials and Methods

This pilot project was run at SB HOME, Renaissance School of Medicine’s student-run free medical clinic in Patchogue, New York. Protocols and procedures were approved by the Stony Brook Institutional Review Board and deemed exempt (Study # IRB2020-00636).

Survey questions focused on patient demographics, telehealth utilization, device ownership, barriers to appointment access, and patient-perceived attitudes toward telehealth, such as comfort with virtual visits and confidence in telemedicine platforms supporting their health care. Likert-scale questions to specifically assess comfort and confidence were adapted from the work of Benavent et al. who also investigated patient perceptions toward telemedicine at an institutional level.^8^ The REDcap (Research Electronic Data Capture) survey platform was used to electronically administer and store survey responses.^9^

Surveys were administered bimonthly from May 2022 to August 2023 when the clinic was in session. Patients (*n* = 100) were approached by medical students trained in survey administration. Patients were educated about study involvement, and verbal consent was obtained prior to survey participation. Waiting room time was leveraged, and patients were given the option to take surveys in either English or Spanish on an iPad with SB HOME interpreters available on site as needed for clarification. Spanish surveys were translated from the English format and independently verified for accuracy and clarity by two SB HOME translators. Survey completion time ranged between 5 and 7 min.

Descriptive statistics were applied to the patient responses. A chi-squared analysis was performed on Python to investigate differences in patient responses based on preferred language, English versus Spanish, for various parameters.

## Results

Overall, survey respondents identified as female (54%), male (44%), or other (2%). Respondents ethnically identified as Hispanic or Latino (68%). Most respondents (52%) were within the 40–60 age group and completed high school and/or some college (62%). For the English-speaking group, 90% had completed high school and/or college, whereas only 62.7% of Spanish speakers had done the same (Table 1). While English speakers came from a wide variety of zip codes, >50% of Spanish speakers were found to come from primarily three zip codes in the area: Brentwood 11717 (25%), Central Islip 11722 (17%), and Patchogue 11772 (15%).

**Table 1.** Demographic Profiles of Patients in the English- and Spanish-Speaking Groups

	English (*n* = 40)	Spanish (*n* = 60)	Total (*n* = 100)
Gender/ethnicity (%)			
Female	60	50	54
Male	40	46.7	44
Transgender	0	1.7	1
Gender nonconforming	0	1.7	1
Hispanic and/or Latino (%)	20	100	68
Age (%)			
Under 18	2.5	0	1
18–30	12.5	3.3	7
30–40	12.5	20.0	17
40–60	50	53.3	52
60–70	12.5	15	14
70+	10	8.3	9
Education (%)	(*n* = 40)	(*n* = 59)	(*n* = 99)
No schooling	0	13.6	8.1
Nursery to eighth grade	5	13.6	10.1
Some high school	5	10.2	8.1
High school	37.5	39	38.4
Undergraduate or higher	52.5	23.7	23.7

Awareness and utilization of SB HOME’s telehealth option were low among both English and Spanish speakers. Of all survey patients, 29% were aware that telehealth was offered at SB HOME. Telehealth utilization was 20% across all participants.

Differences in travel times were not observed between groups, with 52% of all respondents traveling between 10 and 30 min to get to clinic (*p* = 0.67, *χ*^2^ = 0.81). Participants were significantly more likely to drive themselves to clinic versus carpool/rideshare (*p* = 0.0014, *χ*^2^ = 10.16). However, Spanish speakers more likely to rely on carpooling/rideshare options (47.2% vs. 15.4%) rather than driving independently (52.8% vs. 84.6%) to clinic when compared with English speakers.

The use of childcare services or missed workdays was also assessed. While most participants stated they had children (78%), only 1% stated they paid for childcare to go to the clinic.

Factors influencing access to telehealth were assessed. Overall, respondents varied in their ownership of electronic devices, smartphones (78%), computers (38%), and tablets (25%), with smartphones being the most utilized device. However, across all modalities, Spanish speakers were significantly less likely than English speakers to own a smartphone (68.3% vs. 92.5%), a computer (20% vs. 65%), or a tablet (13.3% vs. 42.5%; *p* = 0.046, *χ*^2^ = 6.15). Albeit not significant, Spanish speakers were also less likely to have a private space to complete a virtual visit (76.7% vs. 97.5%). Similarly, while all English speakers reported reliable internet access, only 66.7% of Spanish speakers reported the same (Table 2).

**Table 2.** Differences in Device Ownership and Appointment Access Across English- and Spanish-Speaking Groups

	English (*n* = 40)	Spanish (*n* = 60)	Total (*n* = 100)	
Device ownership				
Own a smartphone (%)	92.5	68.3	78	
Own a computer (%)	65	20	38	
Own an electronic tablet (%)	42.5	13.3	25	*p* = 0.046^*^ χ^2^ = 6.15
Appointment access				
Have a device with video access (%)	95	65	77	
Have a private space at home or work where they could complete a virtual visit (%)	97.5	76.7	85	
Reliable internet access (%)	100	66.7	80	*p* = 0.85 *χ*^2^ = 0.33
Transport to clinic				
Carpool/rideshare	15.4	47.2	33.7	
Drive independently	84.6	52.8	66.3	*p* = 0.0014^*^ χ^2^ = 10.16

^*^ *p* < 0.05.

Comfort and confidence levels with respect to telehealth access and usage were also evaluated. Both groups expressed that telehealth would perform dependably and accurately (Likert ≥3, English: 85% vs. Spanish: 68%), but had decreased confidence that telehealth visits would be the same as in-person visits (Likert <3, English: 50% vs. Spanish: 52%). Spanish speakers were significantly less comfortable using technology like a smartphone or a tablet compared with English speakers (Likert <3, English: 8% vs. Spanish: 27%, *p* = 0.0033, *χ*^2^ = 17.77). They were also significantly less comfortable with using technology like a smartphone or tablet for their health care (Likert <3, English:13% vs. Spanish: 30%, *p* = 0.03, *χ*^2^ = 12.40) (Table 3).

**Table 3.** Likert-Scale Assessment of Comfort and Confidence Levels with Telehealth Access and Usage Among English- and Spanish-Speaking Groups

	English (*n* = 40)	Spanish (*n* = 60)	
How comfortable are you with using technology like a smartphone or a tablet? (x̄)	0: 0%	0: 5.1%	*p* = 0.0033^*^ χ^2^ = 17.77
	1: 2.5%	1: 13.6%	
	2: 5%	2: 8.5%	
	3:0%	3: 13.6%	
	4:17.5%	4: 22.0%	
	5: 75%	5: 37.3%	
How confident are you that technology like a smartphone or tablet can support your access to health care? (x̄)	0: 0%	0: 5%	*p* = 0.27 *χ*^2^ = 6.42
	1: 5%	1: 8.3%	
	2: 5%	2: 13.3%	
	3: 12.5%	3: 18.3%	
	4: 17.5%	4: 28.3%	
	5: 60%	5: 26.7%	
How comfortable are you with using technology like a smartphone or tablet for your health care? (x̄)	0:0%	0:6.7%	*p* = 0.03^*^ χ^2^ = 12.40
	1:7.5%	1: 10%	
	2:5%	2:13.3%	
	3: 17.5%	3: 15%	
	4: 17.5%	4: 20%	
	5: 52.5%	5: 35%	
How confident are you that Telehealth technology will perform dependably and accurately? (x̄)	0: 2.5%	0: 8.3%	*p* = 0.44 *χ*^2^ = 4.81
	1: 5%	1:8.3%	
	2: 7.5%	2: 15%	
	3: 20%	3: 23.3%	
	4: 25%	4: 16.7%	
	5: 40%	5: 28.3%	
How confident are you that a Telehealth visit is the same as an in-person visit? (x̄)	0: 7.5%	0: 13.3%	*p* = 0.95 *χ*^2^ = 1.13
	1: 17.5%	1: 16.7%	
	2: 25%	2: 21.7%	
	3: 17.5%	3: 16.7%	
	4: 12.5%	4: 15%	
	5: 20%	5: 16.7%	

“0” for least comfort/confidence and “5” for most confidence.

^*^ *p* < 0.05.

## Discussion

Since the COVID-19 pandemic, telehealth has risen in usage, leading to a fundamental change in how providers practice medicine.^10^ In the United States, while 0.1% of Medicare primary care appointments were conducted with telehealth prior to the pandemic, this number rose to 44% in April 2020.^11^ At SB HOME, the telemedicine program was launched during the COVID-19 pandemic to promote continuity of care in the absence of in-person visits.

While in-person care has been a reliable option for patients at SB HOME, it has posed its own share of obstacles. Obstacles to in-person office visits include transportation costs, child care, and work absenteeism.^3^ These costs are often disproportionately experienced by vulnerable populations. For instance, among patients who visited our clinic, Spanish speakers were more likely than English speakers to rely on ridesharing options (47.2% vs. 15.4%) and less likely to drive independently (52.8% vs. 84.6%) to clinic. Telehealth adoption would reduce these costs, significantly decreasing the burden felt by vulnerable populations. However, our study supported the trend of telehealth underutilization since its inception, with only 20% of clinic patients overall found to have completed a telehealth visit.

Effectively adopting telehealth into the health care system requires a comprehensive evaluation of the patient population being served. Race, socioeconomic status, and health are interconnected. These factors need to be considered for equitable adoption of telehealth across various demographics. At our clinic, 68% of survey respondents identified as Hispanic or Latino, reflecting the demographic majority. In one study that evaluated the use of telemedicine in outpatient oncology care, Spanish-speaking patients had 29% decreased odds of utilizing telemedicine compared to other non-English-speaking patients.^12^ Beyond language, telemedicine was also found to be used at a lower rate among individuals residing in low-income zip codes, those covered by Medicaid, and across vulnerable populations.^12–14^ At our clinic, >50% of Spanish speakers came from three zip codes (Brentwood, Central Islip, and Patchogue) noted for high social vulnerability on Long Island, NY, according to the CDC.^7^ Low socioeconomic status has been shown to be linked to reduced access to quality health care, with 100% of SB HOME patients being uninsured. The absence of insurance has been linked to adverse outcomes, including higher mortality.^5^

Identifying and addressing the barriers to telehealth that vulnerable populations face is key to an equitable implementation of telehealth. Our survey revealed that technological access was a major barrier resulting in the low adoption of telehealth within the Hispanic/Latino population. Spanish speakers were less likely than English speakers to own a smartphone, computer, or tablet (*p* = 0.046). To effect true change, closing this gap in device ownership must be complemented by an equal rise in internet access. Nationally, according to the Pew Research Center, over 91% of Hispanic U.S. adults reported they had a smartphone, while only 75% reported having home broadband.^15^ Within our surveyed population, all English speakers reported reliable internet access; only 66.7% of Spanish speakers reported the same.

Beyond technological needs, Spanish-speaking patients were also less likely to report a private space to conduct their virtual visits than English-speaking patients (76.7% vs. 97.5%). Households with a lower socioeconomic status are often large with little to no room for privacy from children.^16^ This can pose a significant barrier to the clear communication of sensitive medical information.^3^

Patients generally expressed confidence in the ability of telehealth to perform dependably and accurately. However, Spanish speakers were significantly less comfortable using the technology associated with telehealth visits, such as smartphones or tablets, when compared with English speakers (*p* = 0.0033) and less comfortable using it for their health care (*p* = 0.03). Multiple studies have demonstrated a significant lack of confidence in telehealth by the underserved populations.^16,17^ This has been attributed to various reasons, such as issues with consistent internet connectivity, a higher level of distrust of the technology, information privacy concerns, as well as a fundamental lack of technology literacy stemming from low device ownership.^16,17^

Factors impeding telehealth utilization were identified on a systems level as well. Awareness of our clinic telemedicine option was limited, <30%, thus reflecting the need to promote the telehealth option at a clinic level. Limited awareness of the telehealth option at the clinic could in part explain its underutilization. Among patients unaware of it or who chose not to use telehealth despite access, assessing their attitudes toward telehealth would still be valuable in informing future telehealth improvement interventions. Furthermore, the platform utilized for telemedicine encounters was Microsoft Teams. Not only is this platform not specifically designed for this purpose, but there was also no formal training provided to patients to facilitate their utilization of it.

Based on this study, decreased device ownership and a lack of comfort using telehealth were major barriers to telehealth for the Spanish-speaking population. Raising comfort levels can be accomplished through a variety of means. Waiting room time can be leveraged to increase technological literacy. Our patient population demonstrated a baseline literacy, with 62% having completed high school and/or some college that we could build upon. Specific measures to build digital literacy will include tutorials on device operation and accessing and navigating the telehealth platform. Next, partnerships with telehealth platforms with easy-to-operate interfaces for both patients and interpreters can streamline visits, as success has been reported in similar student-run free-health clinics.^18^ Given that smartphones were the most highly utilized device, selecting platforms that are also mobile friendly would be most effective. Finally, while patient education may help raise confidence in telehealth as an alternate medium to patient–provider visits, federal and state interventions to improve broadband access and device ownership may also be needed. Awareness for government-funded broadband connectivity programs and smartphone distribution programs will be raised to accomplish this goal. Similarly, awareness will be raised for telehealth by modifying the appointment scheduling workflow to include offering telemedicine for appropriate encounters.

This study is limited in its single-center-based evaluation. Adopting this survey-based model to assess telehealth perceptions at similar free-health clinics would improve its external validity and provide a more comprehensive dataset with diverse patient demographics. The study groups were created on the basis of language as appropriate for our clinic demographic, given that language of choice could readily be leveraged to target interventions if barriers were discovered. However, using a multivariate analysis to account for other social determinants of health could improve the generalizability of this study. The study is also limited to the patient perspective. Including provider-based surveys to gauge their perceptions of the obstacles to telehealth would augment our understanding of it. Finally, not every patient in the clinic waiting room was amenable to survey participation that could have incorporated some level of sampling bias. However, the demographics of the survey leaned primarily Hispanic/Latino (68%), which is representative of the clinic majority.

## Conclusion

Telehealth offers the potential to overcome significant barriers to health care faced by vulnerable populations. However, it is currently a privilege limited to those who can access it. Significant disparities in telehealth access have been demonstrated between Spanish and English speakers within this single-center study. Increasing device ownership among Spanish speakers, improving internet coverage, and launching survey-directed interventions, such as technology literacy sessions to improve technological comfort, can bring an equitable adoption of telehealth to the patient demographic that needs it the most.

## Abbreviations Used

CDC: Centers for Disease Control and Prevention
COVID-19: Coronavirus disease of 2019
NY: New York
REDcap: Research Electronic Data Capture
SB HOME: Stony Brook HOME
