# Supplementary material for: Exploring Barriers Toward Telehealth in an Underserved, Uninsured Patient Population
Source: Telemed Rep. 2024 Aug 19;5(1):263–8. doi: 10.1089/tmr.2024.0036 (PMC11347875; doi:10.1089/tmr.2024.0036)
Supplement: Supplementary Data S1 [file tmr.2024.0036_supplementaldata.pdf]

Patient Survey

Please take a few minutes to complete this survey. Your responses are valuable and much appreciated.

Do you prefer to speak in English or Spanish?	◯ English<br>◯ Spanish
Edad	◯ Menor de 18 años<br>◯ 18-30<br>◯ 30-40<br>◯ 40-60<br>◯ 60-70<br>◯ 70+
Género	◯ Masculino<br>◯ Femenino<br>◯ Mujer transgenéro<br>◯ Hombre transgenéro<br>◯ Género no conforme/variante<br>◯ No enlistado<br>◯ Prefiero no contestar
Raza (Marque todo lo que corresponda)	☐ Blanco<br>☐ Negro o Afroamericano<br>☐ Índio Americano o Nativo de Alaska<br>☐ Asiático<br>☐ Nativo Hawaiano u Otro Isleño Del Pacifico<br>☐ Birracial<br>☐ Multirracial
Etnia	◯ Hispano o Latino<br>◯ No Hispano o Latino
Codigo Postal	______________________
¿Cuál es su nivel más alto de educación?	◯ Sin escolaridad<br>◯ Guarderia hasta octavo grado<br>◯ Alguna escuela secundaria<br>◯ Escuela secundaria<br>◯ Pregrado (licenciatura) o superior
¿Cuánto tiempo se tarda en llegar a la clínica?	◯ Menos de 10 minutos<br>◯ 10 a 30 minutos<br>◯ 30 minutos a 1 hora<br>◯ Mas de 1 hora
¿Viajó desde casa?	◯ Si<br>◯ No
¿Tomó transporte público para llegar a la clínica?	◯ Si<br>◯ No
¿Cuánto fue su tarifa, ida y vuelta? Redondear al dólar más cercano.	______________________

Si usted no tomó transporte público para llegar a la clínica, usted..	◯ Manejó<br>◯ Compartió vehículo<br>◯ Usó algún tipo de viaje compartido (Taxi, Uber, Ascensor)<br>◯ Caminó
¿Ha faltado al trabajo para venir a la clínica?	◯ Si<br>◯ No
¿Tiene hijos?	◯ Si<br>◯ No
¿Tuvo que pagar por el cuidado de niños para venir a la clínica?	◯ Si<br>◯ No
¿Usted sabe que se ofrece telesalud en SB HOME?	◯ Si<br>◯ No
¿Alguna vez ha completado una cita de telesalud?	◯ Si<br>◯ No
¿Tiene un teléfono inteligente?	◯ Si<br>◯ No
¿Recibió alguna vez un teléfono móvil de parte de SB HOME?	◯ Si<br>◯ No
¿Tiene una computadora?	◯ Si<br>◯ No
¿Tiene una tableta electrónica?	◯ Si<br>◯ No
¿Tiene un dispositivo con acceso a vídeo?	◯ Si<br>◯ No
¿Tiene acceso confiable a Internet?	◯ Si<br>◯ No
¿Tiene un espacio privado en su casa o trabajo donde podría hacer una visita virtual?	◯ Si<br>◯ No

En una escala del 1 al 5, califique las siguientes preguntas (de 0 = menor cantidad a 5 = mayor cantidad)

	0	1	2	3	4	5
¿Qué tan cómodo se siente con el uso de tecnología como un teléfono inteligente o una tableta?	◯	◯	◯	◯	◯	◯

¿Qué tan seguro está de que la tecnología como un teléfono inteligente o una tableta puede respaldar su acceso a la atención médica?	◯	◯	◯	◯	◯	◯
¿Qué tan cómodo se siente con el uso de tecnología como un teléfono inteligente o una tableta para su atención médica?	◯	◯	◯	◯	◯	◯
¿Qué tan seguro está de que la tecnología de telesalud funcionará de manera confiable y precisa?	◯	◯	◯	◯	◯	◯
¿Qué tan seguro está de que una visita de Telesalud es lo mismo que una visita en persona?	◯	◯	◯	◯	◯	◯

Demographics

Age	◯ Under 18<br>◯ 18-30<br>◯ 30-40<br>◯ 40-60<br>◯ 60-70<br>◯ 70+
Gender	◯ Male<br>◯ Female<br>◯ Transgender Female<br>◯ Transgender Male<br>◯ Gender non conforming/variant<br>◯ Not listed<br>◯ Prefer not to answer
Race	☐ White<br>☐ Black or African American<br>☐ American Indian or Alaska Native<br>☐ Asian<br>☐ Native Hawaiian or Other Pacific Islander<br>☐ Biracial<br>☐ Multiracial
Ethnicity	◯ Hispanic or Latino<br>◯ Not Hispanic or Latino
Zip code	__________________________
What is your highest level of education?	◯ No schooling<br>◯ Nursery to eighth grade<br>◯ Some high school<br>◯ High School<br>◯ Undergraduate or higher

Travel to SB HOME

How long does it take you to get to the clinic?	◯ < 10min<br>◯ 10-30 min<br>◯ 30 min-1 hour<br>◯ >1 hour
Did you travel from home?	◯ Yes<br>◯ No
Did you take public transportation to get to the clinic?	◯ Yes<br>◯ No
If yes, how much was your roundtrip fare? Round to the nearest dollar.	________________________
If you didn't take public transportation to the clinic, did you...	◯ Drive yourself<br>◯ Carpool<br>◯ Rideshare (Taxi, Uber, Lyft)<br>◯ Walk
Have you missed work to come to the clinic?	◯ Yes<br>◯ No
Do you have any children?	◯ Yes<br>◯ No
If yes, did you have to pay for childcare to come to the clinic?	◯ Yes<br>◯ No

Awareness

Do you know that telehealth is offered at SB HOME?	◯ Yes<br>◯ No
Have you ever completed a telehealth appointment?	◯ Yes<br>◯ No
Do you own a smartphone?	◯ Yes<br>◯ No
Did you ever receive a mobile phone from SB HOME?	◯ Yes<br>◯ No
Do you own a computer?	◯ Yes<br>◯ No
Do you own an electronic tablet?	◯ Yes<br>◯ No
Do you have a device that has video access?	◯ Yes<br>◯ No
Do you have reliable internet access?	◯ Yes<br>◯ No

Do you have a private space at your home or work where you could do a virtual visit?

◯ Yes
◯ No

On a scale of 1-5, please rate the following questions (from 0 = least amount to 5 = most amount)

	0	1	2	3	4	5
How comfortable are you with using technology like a smartphone or a tablet?	◯	◯	◯	◯	◯	◯
How confident are you that technology like a smartphone or tablet can support your access to healthcare?	◯	◯	◯	◯	◯	◯
How comfortable are you with using technology like a smartphone or tablet for your healthcare?	◯	◯	◯	◯	◯	◯
How confident are you that Telehealth technology will perform dependably and accurately?	◯	◯	◯	◯	◯	◯
How confident are you that a Telehealth visit is the same as an in-person visit?	◯	◯	◯	◯	◯	◯
